# Detection and Severity Assessment of Parkinson’s Disease Through Analyzing Wearable Sensor Data Using Gramian Angular Fields and Deep Convolutional Neural Networks

**DOI:** 10.3390/s25113421

**Published:** 2025-05-29

**Authors:** Sayyed Mostafa Mostafavi, Shovito Barua Soumma, Daniel Peterson, Shyamal H. Mehta, Hassan Ghasemzadeh

**Affiliations:** 1 College of Health Solutions, Arizona State University, Phoenix, AZ 85004, USAhassan.ghasemzadeh@asu.edu (H.G.); 2 Department of Neurology, Mayo Clinic College of Medicine, Mayo Clinic Arizona, Scottsdale, AZ 85259, USA; mehta.shyamal@mayo.edu

**Keywords:** Parkinson’s disease, Gramian angular field, gait, convolutional neural network, Parkinson’s diagnosis and prognosis

## Abstract

Parkinson’s disease (PD) is the second-most common neurodegenerative disease. With more than 20,000 new diagnosed cases each year, PD affects millions of individuals worldwide and is most prevalent in the elderly population. The current clinical methods for the diagnosis and severity assessment of PD rely on the visual and physical examination of subjects and identifying key disease motor signs and symptoms such as bradykinesia, rigidity, tremor, and postural instability. In the present study, we developed a method for the diagnosis and severity assessment of PD using Gramian Angular Fields (GAFs) in combination with deep Convolutional Neural Networks (CNNs). Our model was applied to PD gait signals captured using pressure sensors embedded into insoles. Our results indicated an accuracy of 98.6%, a true positive rate (TPR) of 99.2%, and a true negative rate (TNR) of 98.5%, showcasing superior classification performance for PD diagnosis compared to the methods used in recent studies in the literature. The estimation of disease severity scores using gait signals showed a high accuracy for the Hoehn and Yahr score as well as the Timed Up and Go (TUG) test score (R^2^ > 0.8), while we achieved a lower prediction performance for the Unified Parkinson’s Disease Rating Scale (UPDRS) and its motor component (UPDRSM) scores (R^2^ < 0.2). These results were achieved using gait signals recorded in time windows as small as 10 s, which may pave the way for shorter, more accessible assessment tools for diagnosis and severity assessment of PD.

## 1. Introduction

PD ranks as the second-most common neurodegenerative disorder of the motor system [1], affecting approximately 2–3% of individuals over the age of 65. This amounts to nearly 1 million individuals in North America and more than 20,000 new cases per year in the United States alone [2,3]. The risk of PD increases with age, which can create a significant financial burden on healthcare systems in countries with a large elderly population [4]. PD results in considerable neuropathology that affects many regions of the brain. This results in varied symptoms ranging from motor deficits, such as gait and balance dysfunction or dysphagia, to cognitive deficits such as dementia and psychosis [5].

Gait can be severely affected in people with PD, which reduces their mobility [6,7,8]. Parkinsonian gait is characterized by shuffling steps, a reduced stride length and walking speed, and a limitation in or lack of arm swinging. These can collectively pose an increased risk of falls [9]. Freezing of gait (FOG) is a sudden and temporary halt in walking and is considered another common characteristic of Parkinsonian gait [10]. These symptoms often cause a loss of independence and reduced mobility, making gait analysis essential for PD management and a crucial tool in neurological assessments [11].

The current clinical procedure for diagnosing PD involves a detailed examination of the medical history and motor symptoms. Typical hallmark symptoms of PD include tremors, rigidity, bradykinesia, and postural instability. Clinicians employ an array of standardized scales for diagnosis and severity assessment of PD. These include the UPDRS, the Timed Up and Go (TUG) test, and the Hoehn and Yahr score [12,13]. Brain imaging modalities such as MRI may also serve as complementary diagnosis tools to rule out other causes [14]. Despite their standard use in clinical practice, these clinical assessment tools may lack diagnostic accuracy. Moreover, they can be time-consuming and cumbersome, which can potentially delay early detection. This is important since therapeutic interventions are more effective in the early stages of the disease [15,16].

Gait analysis is an important aspect of the neurological examination for diagnosing PD. This is often conducted in the form of a visual examination to assess features such as stride length, walking speed, postural stability, and arm swing [17]. The visual examination of subjects suffers from several limitations. First, it is limited by inter-subject variability. This can, in turn, lead to subjective interpretations depending on the clinician’s experience. Moreover, these visual examinations are often performed under controlled environments, which may not reflect the variabilities in gait patterns in real-world settings [18]. To address these limitations, the utilization of more objective methods such as using wearable sensor data has become increasingly important. Such data can objectively quantify gait parameters, thus providing an accurate method for identifying subtle abnormalities and monitor disease progression [19].

Several previous studies have applied machine learning models in conjunction with objectively acquired datasets for PD diagnosis and prognosis. These included, but were not limited to, handwriting pattern [20], neuroimaging [21], cerebrospinal fluid [22], serum-derived biomarker [23], and voice [24] data. Furthermore, sensor data were used for objective analyses of gait patterns in individuals with PD. Hausdorff et al. [25] analyzed gait speed characteristics in individuals with PD and controls by applying statistical methods to data acquired using sensors. In another study [26], the same research group used descriptive analyses to compare the gait speed of PD and control participants on level ground and a treadmill.

Rodríguez-Martín et al. [27] applied a machine learning model based on random forests and used IMU sensor data for the automatic detection of freezing of gait episodes and reported a high accuracy. Similarly, Zhao et al. [28] used a recurrent neural network model to investigate the temporal dependencies in gait data and reported improved performance in classifying gait patterns in individuals with PD. In another study, Eskofier et al. [29] used smartphone-based sensors to acquire data and train SVM and decision trees, demonstrating that this approach could differentiate subjects with PD from healthy controls with high accuracy. Another study on the classification of Parkinsonian gait was conducted by Rigas et al. [30]. They applied models based on support vector machines and deep neural networks and reported high precision and recall rates.

Altogether, prior research has shown that favorable results can be achieved for the detection and severity assessment of PD using a breadth of machine learning models and data acquisition techniques. However, many of these datasets may be time-consuming to obtain for each individual. Moreover, in many of the aforementioned studies, the feature extraction techniques used to obtain gait patterns are domain-specific and may not be comprehensive. GAFs are a feature extraction technique that is applied to 1D time series data and offers unique advantages by encoding the temporal dynamics into visually interpretable images [31]. GAFs preserve the temporal order and correlations between data points. This allows GAFs to integrate both spatial and temporal information more effectively than some other feature extraction methods. This unique representation enables the use of advanced image-based techniques, such as CNNs, for improved pattern recognition. Moreover, GAFs can potentially capture subtle structural changes in the time series compared to frequency-based methods, which may lose information about local patterns during transformations [31].

In the present study, we investigated the potential of using GAFs to extract relevant features from gait patterns in subjects with PD. This was used in combination with a CNN model detecting PD. Our approach can be applied to shorter time windows. This has the potential to shorten the time required for the physical examination of subjects with PD. Furthermore, it can be used to extract meaningful features for PD severity assessments.

## 2. Materials and Methods

### 2.1. Gait in Parkinson’s Dataset

The dataset used in this study was obtained from PhysioNet [32]. It is a compilation of data collected in a number of different studies [33,34,35,36]. The origin of the datasets is denoted by *Si*, *Ga*, and *Ju* within the compiled dataset published by PhysioNet [32]. These data were collected at three different laboratories using a similar set of instructions. Some of these datasets also include laboratory measurements obtained under different gait conditions, such as when performing serial 7 subtraction [34] or exposed to different auditory stimuli [35]. These altered gait conditions were not considered for analysis in this study.

**Figure 1:**
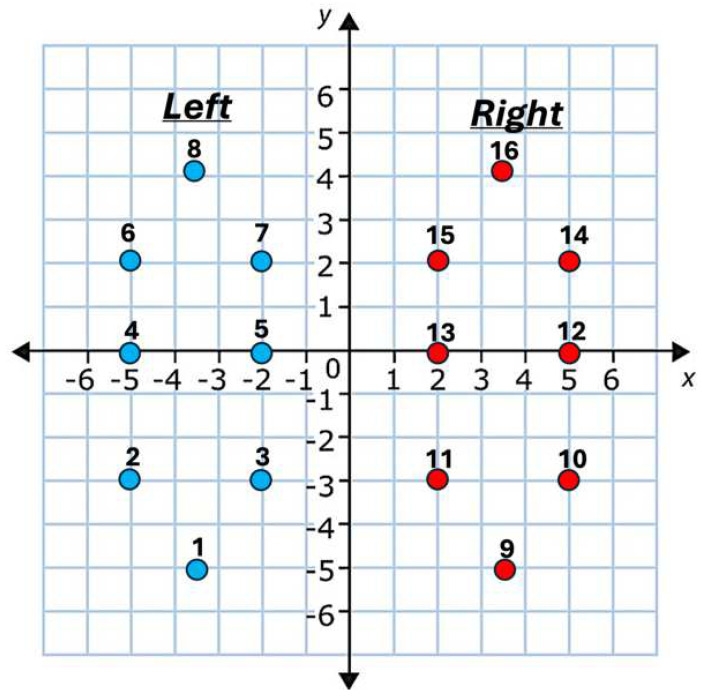
Relative VGRF sensor location under two feet with parallel legs. Scaling is arbitrary.

In this study, our analysis was based on data collected from 91 individuals with PD (mean age ± standard deviation: 66.3 ± 9.4 years; 58 male participants (64%)) and 72 healthy control subjects (mean age ± standard deviation: 63.6 ± 8.6 years; 39 male participants (54%)). Vertical Ground Reaction Force (VGRF) measurements were collected from 8 sensors placed under each foot (a total of 16 channels from both feet) during a walking trial of approximately 2 min, with a sampling frequency of 100 Hz [32]. In this study, we used the sum of the VGRF measurements from each foot as a marker of gait and did not consider individual sensor readings. Figure 1 shows the relative location of the VGRF sensors under the two feet.

**Figure 2:**
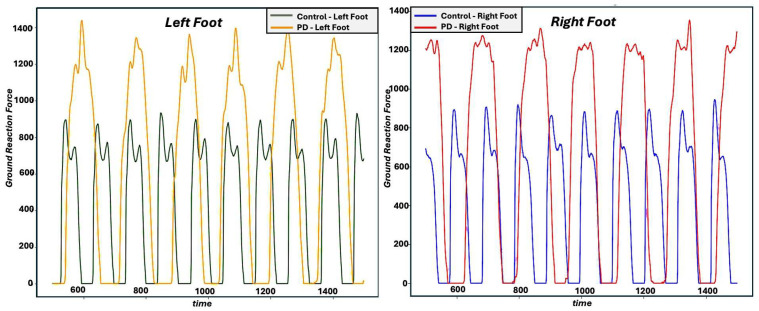
Sample VGRF recording of sum of eight sensors for both feet. Blue and green lines represent control subjects; red and yellow lines represent PD participants.

PD can affect gait in a number of ways. For example, the continuous nature of gait can be influenced, causing smaller, more variable, and more asymmetric steps, with altered timing. In addition, gait can be impacted in acute ways, with festination and freezing being common effects, particularly in later stages of the disease. Figure 2 shows the gait signals for two subjects: a control subject and a PD participant in a 10 s time window (left and right panel for left and right foot, respectively). It can be observed that the control subject completed more gait cycles than the PD participant, i.e., a slower gait for the PD participant. We can also observe more variability in the gait patterns associated with the PD participant compared to the control subject. In addition, we can observe some asymmetry between the left and right gait patterns in the PD participant, whereas the patterns for the control subject appear more symmetric between the two feet.

### 2.2. Gramian Angular Fields

**Figure 3:**
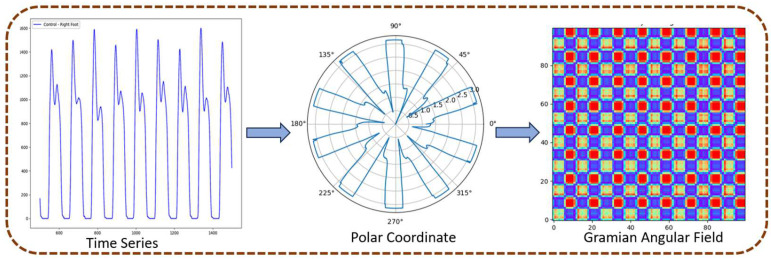
Steps in the transformation of 1D time series data to its corresponding 2D GAF image.

GAF transformation [37,38,39] is an efficient and frequently adopted imaging technique that is used to transform a 1D signal into a 2D image. In the present study, we used this technique to transform 1D time series gait signals into 2D images. To this end, GAFs were used to first normalize the time series data by scaling it to a defined range, typically between −1 and 1, to preserve consistency. Subsequently, the GAFs transform the normalized 1D time series into a polar coordinate system [38]. This encoding process into the polar coordinate system is bijective and preserves the temporal dependency [39,40,41]. Subsequently, a Gram matrix is constructed using trigonometric functions, which captures the time-dependent correlations between data points. This matrix is then represented as a 2D image, which serves as a graphical representation of the input time series while maintaining both the temporal structure and dynamic characteristics of the time series. Figure 3 depicts the steps involved in the transformation of a sample gait pattern time series into its associated GAF image.

In mathematical terms, suppose *s*(*t*) is a time series with *m* number of samples, as shown in Equation (1):

s(t) = [t_1_, t_2_, t_3_, t_4_ … tₘ] (1)

Subsequently, s(t) is scaled to a range of [−1, 1] using Equation (2). This normalization ensures optimal algorithm performance and minimizes the risk of bias [39,40,41].

(2) $$\tilde{t_{i}}=(s(t_{i})-\min(s(t))+\max(s(t))-(s(t_{i}))/\max(s(t))-\min(s(t)))$$

The scaled samples are then transformed into a polar coordinate system using Equation (3):

(3) $$\theta_{i}=\cos^{-1}(\tilde{t_{i}}),\mathrm{where}-1\leq \tilde{t_{i}}\leq 1$$

rᵢ = i/m     i ϵ m

where θᵢ and rᵢ are the inverse value of the cosine function and the radius of *tᵢ* in the polar coordinate system, respectively. Since samples of *s*(*t*) have been scaled to the [−1, 1] range, the cosine angle varies in the range of [0, π]. Subsequently, sample point correlations are measured by the trigonometric functions and are employed to construct a Gram matrix as shown by Equation (4). As such, GAFs transform the 1D time series to a 2D image using a sequence of scaling the data, transforming the coordinates, and applying trigonometric functions.

(4) $$\mathrm{GAF}=\left[\begin{array}{ccc} cos(\theta ₁+\theta ₁) & \ldots & cos(\theta ₁+\theta ₘ)\\ \vdots & \ddots & \vdots \\ cos(\theta ₘ+\theta ₁) & \ldots & cos(\theta ₘ+\theta ₘ) \end{array}\right]$$

### 2.3. CNN Model Architecture

The self-learning capability of CNNs has shown promise for tackling many problems in biomedical signal and image processing [42]. Importantly, CNNs are able to automatically extract meaningful features from input images without manual extraction of multiple features by performing a series of convolution operations using kernel functions [43]. In the present study, we used two CNN models to (i) classify normal and PD gait signals, as defined by their corresponding GAF images; and (ii) estimate four clinical scores: Hoehn and Yahr, UPDRS, UPDRSM, and TUG scores, using gait-generated GAF images as the input.

**Figure 4:**
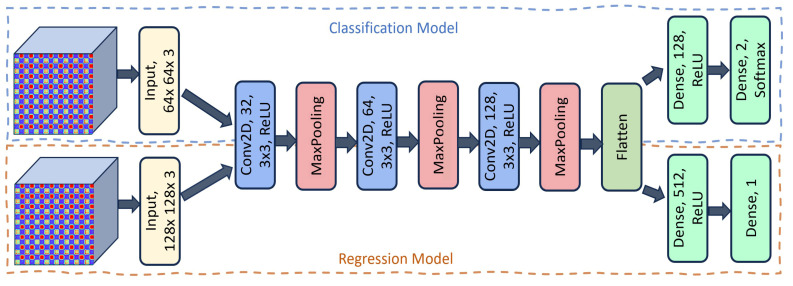
Architecture of CNN model for classification and estimation of clinical scores using GAF images.

The architectures of the two models for classification and regression are illustrated in Figure 4. The classification model takes a 64 × 64 × 3 image as input and uses multiple convolutional layers. Each convolutional layer has a kernel size of 3 × 3 and ReLU as the activation function. This improves the model’s ability to learn non-linear patterns by making the network sparse and enhancing the computational efficiency [43]. After each convolutional block, a max-pooling (MP) layer is applied to reduce the dimensionality and aggregate the most prominent features. The network progressively increases the number of filters (from 32 to 128) across the layers, allowing for more complex feature extraction. Once the convolutional feature maps have been processed, they are flattened and passed into fully connected (FC) layers. Finally, a dense layer with *Softmax* activation outputs the final prediction for the classification of the two classes (control vs. PD).

For the estimation of clinical scores, the architecture follows a similar structure but operates on 128 × 128 × 3 input images. This model also consists of three convolutional layers with ReLU activations and max-pooling layers. Finally, a fully connected layer with 512 neurons is used, followed by the output layer, which contains a single neuron for prediction of the continuous-valued score.

We used the Adam optimizer with an initial learning rate of 0.001. This was adjusted using a learning rate scheduler during training to prevent overfitting and improve convergence. The learning rate was reduced by a factor of 10 if the validation loss did not improve after a predefined number of epochs. In order to avoid overfitting, we applied a dropout rate of 0.5 in the fully connected layers and L2 regularization on the weights with a coefficient of 0.0001.

Our proposed architecture was designed to leverage the convolutional layers effectively for GAF images, capturing both local and global dependencies in gait dynamics. This structure allows for the effective extraction of features present in GAF images that reflect an altered Parkinsonian gait. Alternative, more complex architectures may overfit the image dataset due to excessive complexity, while less complex networks may underperform in feature extraction.

### 2.4. Outline of Proposed Method

**Figure 5:**
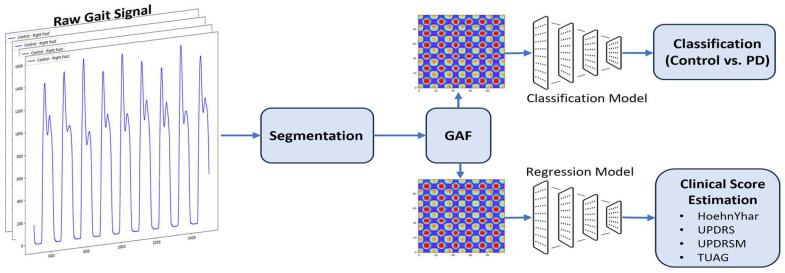
Outline of the proposed method for the classification of PD gait and estimation of clinical scores.

The outline of our proposed system is depicted in Figure 5. Our approach utilizes a CNN architecture to analyze raw gait signals for two primary tasks: classification and clinical score estimation. The raw gait signals are first segmented into time window intervals with varying lengths, ranging from 10 s to 60 s, in 10 s increments. We introduced a 20% window overlap. We hypothesized that such time intervals will capture the essential gait dynamics required for diagnosing and quantifying the severity of the disease. These segments are then transformed into 2D images using GAFs, which encode the temporal dependencies of the time series data in the form of images. This transformation allows the system to leverage the strength of CNNs in image-based feature extraction. This improves the models’ ability to classify and predict clinical scores from the gait data.

The architecture of the two parallel models for the classification and estimation of clinical scores was discussed in the previous subsection. The classification model processes the GAF-transformed images to classify subjects as either PD patients or healthy controls. The regression model follows a similar CNN structure but was designed to estimate clinical scores such as Hoehn and Yahr, UPDRS, UPDRSM, and TUG scores.

The proposed method was implemented in *Python version 3.10.9* using *NumPy* and *SciPy* for data processing, *PyTS* for Gramian Angular Field transformation, and TensorFlow with Keras for CNN development. *PyTS* was used to convert gait signals into images. *Scikit-learn* was employed for dataset splitting and performance evaluation.

## 3. Results

### 3.1. Participants’ Demographics

Table 1 provides the demographic characteristics of the control and PD groups. The study included 72 control subjects (39 males and 33 females) and 91 PD subjects (58 males and 33 females). The gender distribution between the two groups was not significantly different (*p* = 0.282). The mean walking speed was significantly lower in the PD group compared to the control group, with a mean speed of 1.04 m/s (SD = 0.21) in the PD patients versus 1.24 m/s (SD = 0.16) in the controls (*p* < 0.001). Additionally, the difference between the mean ages of the two groups was not statistically significant (*p* = 0.058).

The pool of participants with PD were also evaluated using four clinical measures. The Hoehn and Yahr score, a measure of disease progression, showed that the majority of patients were in stages 2 (54 patients) and 2.5 (27 patients), with 10 patients in stage 3. No subjects were categorized into stages 0, 1, 4, or 5. The UPDRS scores ranged from 13 to 70, with a mean score of 31.6 (SD = 11.8). The motor sub-scores of the UPDRS (UPDRSM), which specifically assesses motor symptoms, ranged from 5 to 44, with a mean score of 19.3 (SD = 7.6). Finally, the TUG test, which is used to assess timing of mobility, showed that PD patients had a mean completion time of 12.1 s (SD = 3.9), with the times ranging from 7.27 to 36.34 s.

### 3.2. Control vs. PD Classification

**Figure 6:**
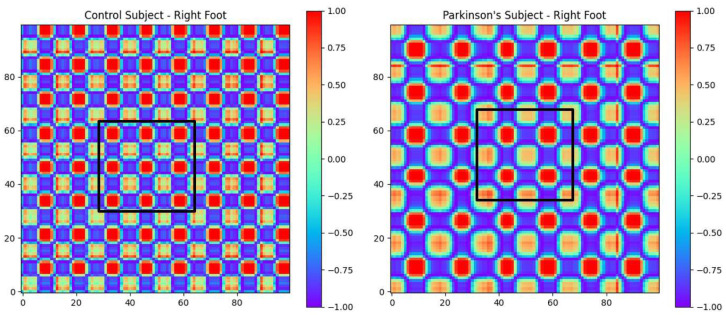
GAF image of gait pattern over 10 s for a control subject (**right**) and PD participant (**left**). The black squares in both images are the same size.

Figure 6 illustrates the GAF images corresponding to a 10 s time window of two subjects: one from the control group and one PD participant. Based on the fact that the black squares in the two images are the same size, it can be observed that, in the control subject image, the square covers a larger number of repeated patterns (i.e., gait cycles) compared to the image for the participant with PD over the same time period. Moreover, the repeated gait patterns were more uniform in shape for the case of the control subject, but appeared more heterogenous for the PD participant. Both of these observations are in line with our understanding of the gait patterns in PD patients, who exhibit slower gait patterns with higher levels of variability [36]. In this study, our general hypothesis was that these patterns that are present in GAF images can capture the gait characteristics of participants with PD and can be used to make deductions about disease diagnosis and clinical biomarkers of Parkinsonian gait.

The two-dimensional encoding of the GAF images captures both the temporal dependencies and amplitude variations in the gait cycle. This is accomplished in such a way that the phase relationships between successive signal values is preserved. This allows this modeling technique to encode patterns related to stride variability, asymmetry, and frequency, which are known hallmarks of Parkinsonian gait. CNNs can effectively learn spatial patterns within GAF images, enabling them to detect subtle differences between healthy control and PD gait signals. Early layers of the CNN detect local textures and edges that correspond to stride-to-stride fluctuations in gait dynamics. Deeper layers learn higher-order spatial representations, capturing irregular gait cycles, decreased rhythmicity, and postural instability, which are all critical in detecting PD.

**Figure 7:**
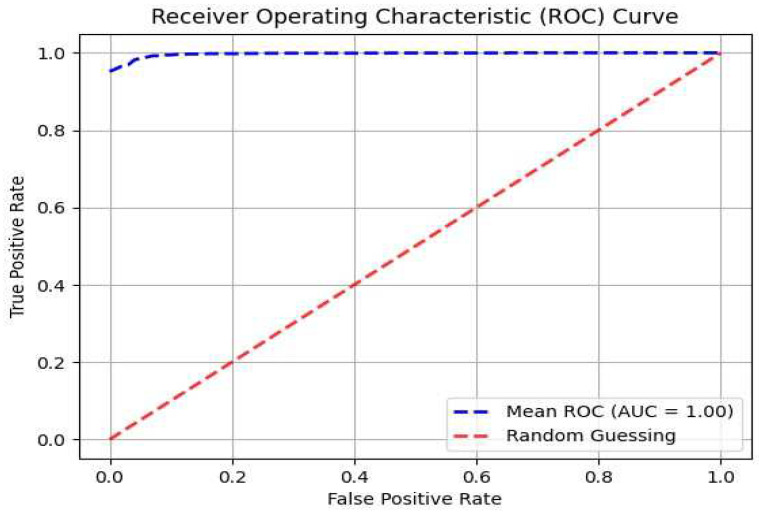
ROC curve for classification of control subjects vs. PD participants. ROC area under the curve = 1.0.

The CNN model was used to classify control subjects vs. PD participants using GAF images of the gait patterns as the input. We achieved a classification accuracy of 98.6%, TPR of 99.2%, and TNR of 98.5% for all the time windows in the 10–40 s window range using 10-fold cross validation. Since our dataset contains data collected in three different laboratories, our cross-validation approach ensures that our developed model is externally validated. Gender-specific models for the male and female sub-populations to classify control subjects vs. PD participants resulted in similar classification performances (male subgroup: ACC = 98.4, TPR = 99.1, TNR = 98.6; female subgroup: ACC = 98.5, TPR = 99.2, TNR = 98.5), indicating that there was no statistically significant difference between the two subgroups (5% significance level). The classification accuracy dropped to 95.2% for time windows larger than 40 s. We postulate that this may be due to the reduced resolution in the GAF image in larger window sizes since the number of repeated gait patterns increases with window size in a given image. The area under the receiver operating characteristic (ROC) curve for our binary classifier was measured to be 1.0. Figure 7 provides the ROC curve for our binary classifier using 10 s time window GAF images.

**Figure 8:**
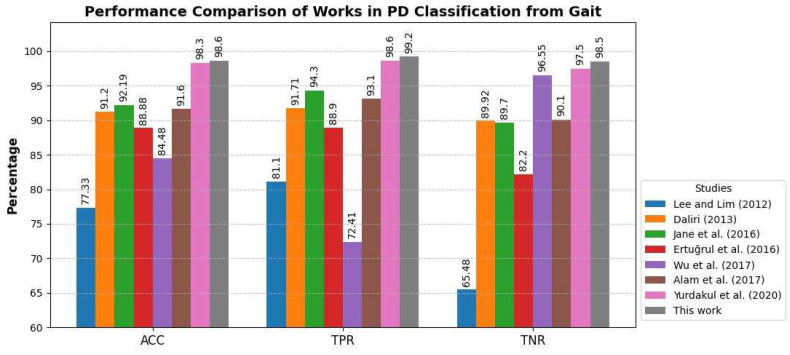
Binary classification performance using gait patterns in studies in the past decade [44,45,46,47,48,49,50] compared to the present study: accuracy (ACC), true positive rate (TPR), and true negative rate (TNR).

To assess the robustness of our modeling approach to random electrical or mechanical noise, we conducted additional experiments by introducing random Gaussian (white) noise to the original gait signals at two different levels: low (σ = 0.01) and medium (σ = 0.05), where σ represents the standard deviation of the added noise. Under low noise conditions, the classification accuracy (ACC) remained at 98.5%, with a true positive rate (TPR) of 99.2% and a true negative rate (TNR) of 98.5%. Even under medium noise conditions, the performance remained highly stable, with an ACC of 98.4%, TPR of 99.0%, and TNR of 98.3%. These findings confirmed that the proposed model is robust to small and moderate levels of noise, a crucial factor given the inherent variability in gait signals recorded from wearable sensors. Furthermore, despite the addition of noise, our approach continued to outperform the methods used in prior studies (Figure 8).

In order to investigate the effect of individual sensor performance (compared to the average of all VGRF sensors), we conducted an ablation study in which we removed VGRF data from four sensors for each foot (#2, 3, 6, and 7 on right foot and #10, 11, 14, and 15 on left foot in Figure 1), while maintaining data for the remaining four sensors. Our results for the classification of PD vs. control participants indicate an accuracy rate of 98.5%, TPR of 99.1%, and TNR of 98.5%. A comparison using the Wilcoxon signed-rank test showed no significant difference at the 95% significance level between these results and those obtained using eight sensors on each foot.

The class distribution in the dataset exhibited a mild imbalance, with a PD-to-control participant ratio of approximately 1.3:1. Given the limited severity of this imbalance and the absence of performance degradation or class bias in the initial model evaluations (accuracy, true positive rate, and true negative rate), explicit imbalance correction was not originally applied. To assess the potential impact of this class distribution on model performance, we conducted additional experiments using the Synthetic Minority Oversampling Technique (SMOTE) to balance the training data. The resulting classification metrics—accuracy of 98.6%, TPR of 99.0%, and TNR of 98.5%—were consistent with those obtained using the original dataset, indicating that the slight imbalance had a negligible effect on the classifier’s performance.

Numerous studies have attempted to classify PD vs. control participants based on gait patterns [44,45,46,47,48,49,50] using the same dataset as the one used in our study [32]. We provide a summary of these studies together with a comparison with our results in Figure 8. Our method outperformed those of previous studies on all three measures of classification (accuracy, TPR, and TNR). The Wilcoxon signed-rank test was used to perform a pairwise comparison of the classification accuracy in our study vs. each individual study listed in Figure 8. With the exception of [51], the difference between classification accuracies was significant at the 95% significance level.

### 3.3. Estimation of Clinical Scores

**Figure 9:**
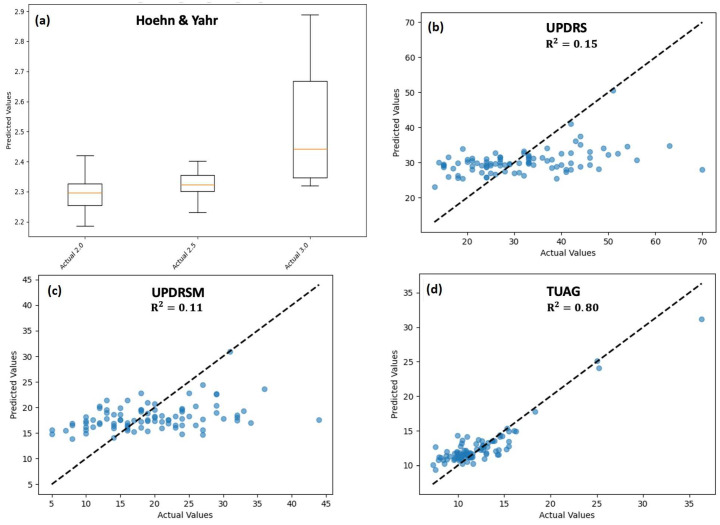
Actual vs. predicted values for four clinical scores estimated using gait pattern signals. Each circle reports aggregated results for one subject. Values reported for (**a**) Hoehn and Yahr score, (**b**) UPDRS (**c**) UPDRS Motor component; and (**d**) TUG. The dotted line represents the perfect fit line.

We used GAF images as the input to our CNN model to estimate four clinical scores. These were the Hoehn and Yahr, UPDRS, UPDRSM, and TUG scores. The R^2^ value, which is a measure of the goodness of fit, was used to compare the actual clinical scores against the values predicted by our model. To calculate this value, the clinical score predicted using several time windows with the same participant was averaged and used as the predicted score for this subject. Table 2 provides a summary of the statistics for the estimation of three clinical scores (UPDRS, UPDRSM, TUG scores) using our estimation approach. The actual vs. predicted estimation results for individual PD participants are presented in Figure 9.

Panel (a) depicts the box plot for the prediction of the Hoehn and Yahr score. Our pool of PD participants only fell into three categories (2.0, 2.5, and 3.0) for this score. Our results showed a mean predicted value of 2.29 (IQR = 2.25–2.31), 2.31 (IQR = 2.30–2.35), and 2.33 (IQR = 2.33–2.65) for the three categories, respectively. A significantly higher prediction variability was observed for the 3.0 class compared to the other two classes. The mean squared error for the entire group of participants was 0.096.

Panels (b) and (c) show the actual vs. predicted values for the UPDRS and UPDRSM scores, respectively. The R^2^ values representing the goodness of fit for these two predictions were 0.15 and 0.11, respectively. The difference between the prediction performances of these two models was not statistically significant (*p* = 0.394). For the UPDRS score (range: 13–70 in our cohort), our estimations had a mean absolute error of 8.35 and a root mean squared error (RMSE) of 10.83. Similarly, for the UPDRSM model (range: 5–44 for our cohort), we obtained a mean absolute error of 5.63 and an RMSE of 7.16. Both of these error bounds are within one standard deviation of the range of values for their corresponding cohort of participants. Gender-specific models for the male and female sub-populations to predict the UPDRS score resulted in similar prediction performances (male subgroup: R^2^ = 0.14; female subgroup: R^2^ = 0.15). There was no statistically significant difference between the two subgroups (5% significance level). We also obtained similar results for the prediction of UPDRSM scores (male subgroup: R^2^ = 0.12; female subgroup: R^2^ = 0.11).

Panel (d) summarizes our findings for the estimation of TUG scores. The associated R^2^ value for this prediction was determined to be 0.80. Our approach estimated the UPDRS score (min–max score range = 7.27–36.34 for our cohort) and achieved a mean absolute error of 1.32 and an RMSE of 1.75. The scores were measured in seconds and are within less than half the standard deviation of the score for our population of PD participants in this study. Gender-specific models for the male and female sub-populations to predict TUG scores resulted in similar prediction performances (male subgroup: R^2^ = 0.78; female subgroup: R^2^ = 0.81). There was no statistically significant difference between the two subgroups (5% significance level).

## 4. Discussion

In the present study, we designed a platform using GAFs coupled with a machine vision technique based on CNNs to diagnose PD and estimate the disease severity using gait pattern signals. Our approach had superior performance in the classification of PD participants vs. control subjects compared to the methods in similar studies over the last decade. Moreover, our classification method outperformed state-of-the-art gait analysis methods in other studies such as Kinect-based systems [51].

Our analysis showed promising results for the estimation of Hoehn and Yahr and TUG scores. The TUG test was designed to evaluate functional mobility and focuses on various aspects of mobility including stability and balance, gait speed and cadence, motor planning and coordination, and freezing of gait. Since all of these were captured in our assessment of gait signals, we expected strong agreement between the TUG score and gait pattern parameters (as observed in the GAF image) in participants with PD. Our results for the estimation of UPDRS and UPDRSM scores were both poor in terms of agreement between the actual score and the scores predicted using our modeling approach (R^2^ < 0.2 for both). It is important to note that the UPDRS and UPDRSM examine numerous aspects of PD symptomology, which are not limited to gait parameters such as tremors. Moreover, the UPDRSM sub-score is heavily weighted towards upper and lower extremity bradykinesia and rigidity. Hence, a poor predictive performance by our platform using gait pattern signals is not surprising. A previous study also found that some of these items were poorly predicted (ICC < 0.4) using the data collected using a Kinect sensor for the upper and lower extremities [52]. Although some aspects of the UPDRSM do focus on gait and balance, only total UPDRSM scores were available in the current dataset, prohibiting us from predicting these sub-scores using the our approach. Future work could enhance the predictive accuracy by integrating multimodal data, such as tremor and bradykinesia assessments or wearable sensor inputs, while also exploring advanced deep learning architectures and domain adaptation techniques to improve model generalization.

One of the key strengths of the present study is that our modeling approach is able to detect PD and make accurate predictions of disease severity using data acquired over time windows as small as 10 s. This can be attributed to the comprehensive nature of our feature extraction technique using GAFs, which allows the temporal dependencies and spatial correlations within the gait signals to be captured. The results of most other studies that examined the present dataset were based on an analysis over the course of 2–3 min [44,45,46,47,48,49,50]. Our proposed modeling approach has important positive implications for clinical assessments, potentially shortening the assessment procedure. We believe our approach can pave the way for shorter visual examinations of patients suspected to have PD, thereby reducing the risk of falls, as well as improving diagnosis efficiency and accessibility.

A limitation of our study is the small number of participants. This hindered our ability to leverage other modeling architectures, such as attention-based neural network approaches. Attention-based models learn global dependencies in data but need significant amounts of labeled training samples, which was not available for our study. Our dataset, while sufficient for CNN-based feature learning, does not meet the scale typically needed for attention mechanisms to achieve optimal performance. Future research could focus on other, more sophisticated architectures as larger data samples become available.

One other limitation of the dataset used in this study was the absence of participants in the early stages of the disease (Hoehn and Yahr score < 2) and those in more severe disease stages (Hoehn and Yahr score > 3), as well as the small number of participants in the different age groups. An important question that remains is whether our modeling approach is able to detect PD in its early stages and if it performs differently across different age groups. Our results indicate that the prediction of disease severity was particularly poor for participants with severe impairments (UPDRS > 30). Another question of interest is whether we can develop tailored models for specific groups of participants to accurately predict the disease severity when we include participants with more severe disease symptoms in our pool of subjects. Furthermore, it would also be interesting to augment our current modeling approach with other data modalities that focus on other aspects of the disease, and investigate whether such an approach can improve the prediction of disease severity.

The findings of this study have potential clinical implications, particularly in the continuous monitoring and management of Parkinson’s disease. By leveraging gait signals transformed into Gramian Angular Fields, the proposed method provides an objective, quantitative approach for assessing motor impairment, which could complement traditional clinical evaluations. This technique could be integrated into wearable sensor systems for remote monitoring, allowing clinicians to track disease progression in real-world settings without requiring frequent in-person visits. Such an approach aligns with the growing emphasis on telemedicine and digital health, offering a practical tool for personalized patient management. Additionally, the predictive capability of this model could be incorporated into AI-driven clinical decision support systems, aiding neurologists by providing automated assessments of motor function. This integration could enhance clinical workflows, reduce subjectivity in gait analysis, and facilitate timely intervention strategies.

## 5. Conclusions

The present study offers a framework for detecting PD from gait signals based on GAF images and deep CNNs. Our model achieved superior performance in all metrics (accuracy, TPR, and TNR). Moreover, our modeling approach was able to accurately estimate the metrics associated with disease severity including the Hoehn and Yahr score as well as TUG score. Our results were achieved using analysis of gait signals in a time window as small as 10 s in length, thus providing a fast and easy tool for detecting and assessing the severity of PD.

## Figures and Tables

**Table 1:** Participants’ demographics.

	Control Subjects	Parkinson’s Subjects	*p*-Value
Number of Participants (Total, Male, Female)	(72, 39, 33)	(91, 58, 33)	0.282
Mean Walking Speed (Mean, SD)	(1.24, 0.16)	(1.04, 0.21)	<0.001
Age (Mean, SD)	(63.6, 8.6)	(66.3, 9.4)	0.058
Hoehn and Yahr Score (0, 1, 2, 2.5, 3, 4, 5)	N/A	(0, 0, 54, 27, 10, 0, 0)	--
UPDRS (Minimum, Maximum, Mean, SD)	N/A	(13, 70, 31.6, 11.8)	--
UPDRSM (Minimum, Maximum, Mean, SD)	N/A	(5, 44, 19.3, 7.6)	--
TUG (Minimum, Maximum, Mean, SD)	N/A	(7.27, 36.34, 12.1, 3.9)	--

**Table 2:** Summary of results for estimation of clinical scores.

Clinical SCORE	Actual Population Statistics (Minimum, Maximum, Mean, SD)	Estimation Statistics (Minimum, Maximum, Mean, SD)	R^2^	MAE	RMSE
UPDRS	(13, 70, 31.6, 11.8)	(23.1, 50.5, 30.3, 3.6)	0.15	8.35	10.83
UPDRSM	(5, 44, 19.3, 7.6)	(13.8, 30.9, 18.1, 2.6)	0.11	5.63	7.16
TUG	(7.27, 36.34, 12.1, 3.9)	(9.4, 31.2, 12.6, 3.0)	0.80	1.32	1.75

## Data Availability

The dataset used in this study is publicly available.
